# Changing Patterns in Infective Endocarditis: A Contemporary Epidemiological Perspective

**DOI:** 10.3390/pathogens15070697

**Published:** 2026-06-30

**Authors:** Vasiliki Rapti, Anna-Pelagia Magiorakos, Efthymia Giannitsioti, Garyfallia Poulakou

**Affiliations:** 1 Third Department of Internal Medicine and Laboratory, School of Medicine, National & Kapodistrian University of Athens, Sotiria General Hospital, 115 27 Athens, Greece; gpoulakou@gmail.com; 2 Independent Researcher, 115 27 Athens, Greece; magiorakosanna@gmail.com; 3 First Department of Propaedeutic and Internal Medicine, School of Medicine, National & Kapodistrian University of Athens, Laiko General Hospital, 115 27 Athens, Greece; gianiemi@hotmail.com

**Keywords:** infective endocarditis, epidemiology, healthcare-associated infection, prosthetic valve endocarditis, cardiac implantable electronic device infection, transcatheter aortic valve replacement, injection drug use, chronic kidney disease, ageing population, *Staphylococcus aureus*

## Abstract

Since its first description in the late nineteenth century, the epidemiology of infective endocarditis (IE) has changed considerably. Once primarily affecting younger individuals with structural heart disease, IE is now increasingly encountered in older patients with multiple comorbidities and frequent healthcare exposure. Population ageing, end-stage renal disease (ESRD), immunosuppression, and injection drug use (IDU) have broadened the pool of susceptible hosts. At the same time, the increasing use of prosthetic valves (PVs), cardiac implantable electronic devices (CIEDs), and transcatheter cardiac interventions has reshaped the clinical spectrum of IE. This epidemiological transition has also been accompanied by shifts in microbiological patterns, with a growing predominance of staphylococci and enterococci, as well as marked geographic and socioeconomic variation in disease burden. This review summarizes the contemporary epidemiology of IE, with an emphasis on the host-, healthcare-, and microbiological factors underlying its evolving clinical profile.

## 1. Introduction

IE continues to represent a serious clinical entity with a disproportionate impact relative to its frequency. Recent global estimates indicate that the burden of IE has risen substantially over the past three decades, with a 34% increase in incident cases, a 29% increase in IE-related deaths, and a 26% increase in disability-adjusted life years [1]. Despite advances in diagnostic and therapeutic strategies, outcomes have improved only modestly, with short-term mortality approaching 17% and rising to approximately 25% in 6 months [2,3].

These trends reflect the evolving epidemiology of IE, which is driven by patient-related risk factors, comorbidities, healthcare exposure, changing microbiological patterns and antimicrobial resistance, as well as regional and socioeconomic differences. In high-income countries with higher socio-demographic indices (SDIs), native valve endocarditis (NVE) now predominantly affects older patients with degenerative valvular disease, multiple comorbidities, and frequent contact with healthcare systems [4,5]. These changes have favored the emergence of healthcare-associated IE, which currently accounts for up to 47% of cases [6]. At the same time, the expanding use of surgical and transcatheter aortic valve replacement (TAVR), along with CIEDs, has increased the incidence of prosthetic valve endocarditis (PVE). The rising prevalence of IDU, particularly in North America and parts of Europe, has further altered the epidemiological landscape of IE, with IDU-associated disease most commonly presenting as right-sided NVE [5]. From a microbiological perspective, *S. aureus*, including methicillin-resistant strains, is the leading cause of both community- and healthcare-associated IE, followed by enterococci and coagulase-negative staphylococci (CoNS), particularly in older and hospitalized patients [2,5].

In contrast, in low- and middle-income countries, NVE continues to affect younger individuals and remains closely related to rheumatic and congenital heart disease, whereas PVE is reported in younger patients and less frequently, potentially owing to limited access to cardiac surgery. Viridans group streptococci predominate, likely reflecting poor oral hygiene and limited access to dental care [5,7]. In these settings, delayed presentation, limited access to transesophageal echocardiography and cardiac surgery, and constrained healthcare resources contribute to more advanced disease, higher complication rates, and increased mortality [5].

The present review provides an overview of the contemporary epidemiology of IE, with particular emphasis on the evolving patient-related characteristics, healthcare-associated factors, and microbiological trends that increasingly influence current clinical practice.

## 2. Host-Related Risk Factors

### 2.1. Ageing and Frailty

Over the past three decades, IE incidence and mortality have progressively shifted toward older age groups, particularly adults aged ≥65 years with multiple comorbidities, with the highest burden observed among individuals aged ≥75 years in high- and middle-SDI countries [1]. Ageing predisposes individuals to infection through immunosenescence, frailty, and cumulative chronic disease burden, resulting in up to a fivefold higher risk of IE compared with the general population [8]. Nevertheless, contemporary registries continue to demonstrate considerable age heterogeneity among patients with ΙΕ, despite the disproportionate burden observed in older adults. Data from the European Endocarditis (EURO-ENDO) Registry reported a mean patient age of 59.25 ± 18.03 years, with 12% of cases occurring in individuals aged over 80 years. Even higher mean ages were described in specific subgroups, including prosthetic valve or repair-associated endocarditis (63.36 ± 16.81 years) and cardiac implantable electronic devices infections (CIEDIs) (66.77 ± 14.11 years) [9]. Similar patterns have been observed in several national registries and observational studies [10,11,12,13], with some suggesting that the incidence of IE among individuals aged ≥80 years has doubled over the past decades.

In older adults, particularly in octogenarians, IE poses distinct diagnostic and therapeutic challenges that are not fully addressed in current guidelines. In this age group, IE frequently manifests atypically, with non-specific constitutional symptoms, attenuated febrile responses, altered mental status, or complications such as heart failure, stroke, septic arthritis, and systemic embolism. Since classic peripheral stigmata are generally absent and routine laboratory findings lack specificity, timely diagnosis requires a high index of clinical suspicion, particularly in the setting of persistent unexplained bacteremia [14]. In addition, comorbidities, such as cardiovascular disease, diabetes mellitus, and malignancy, are prevalent and contribute to frailty, treatment complexity, and higher operative risk, as reflected by elevated EuroSCORE II [15].

Mortality among older adults with IE remains substantial. An analysis of U.S. death certificate data, focusing on individuals aged 65 years and older, demonstrated a temporal pattern in IE-related mortality, with rates increasing until 2004, declining between 2004 and 2018, and rising again through 2020. Across this period, age-adjusted mortality rates per 100,000 were 9.4 (9.3–9.5) among individuals aged 65–74 years and 26.5 (26.3–26.7) among those aged 75–84 years, rising markedly to 79.8 (79.3–80.3) in those aged ≥85 years. Notably, the octogenarians accounted for 42.9% of all IE-related deaths [16]. The initial reduction in mortality is likely attributable to advances in diagnostic accuracy, earlier recognition of IE, improvements in microbiological diagnostics, and greater access to specialized medical and surgical care. In contrast, the subsequent increase may reflect the growing proportion of older and frailer patients with multiple comorbidities, the increasing use of PV and CIEDs, and the rising burden of healthcare-associated infective endocarditis.

Adverse outcomes in older adults are largely attributable to the combined effects of advancing age, comorbidity burden, and surgical interventions. Data from the Spanish GAMES registry, which prospectively enrolled 3120 patients stratified by age (<65 years, *n* = 1327; 65–79 years, *n* = 1291; ≥80 years, *n* = 502), identified age ≥80 years (hazard ratio [HR]: 2.78; 95% confidence interval [CI]: 2.32–3.34), a Charlson Comorbidity Index (CCI) ≥3 (HR: 1.62; 95% CI: 1.39–1.88), and the absence of surgical intervention (HR: 1.64; 95% CI: 1.16–1.58) as independent predictors of mortality. The prognostic performance of the CCI was greater in patients aged <65 years for both in-hospital and 1-year mortality (*p* < 0.001). Interestingly, an age-related discrepancy between surgical indication and actual intervention was noted. Although nearly half of all octogenarians met criteria for surgical intervention, only 21% ultimately underwent the procedure. In contrast, among patients aged <65 years, 68% had a surgical indication and 53% received operative treatment [17]. Similar results were reported in an analysis of 2186 patients with left-sided IE from the Swedish Registry of Infective Endocarditis, which demonstrated a marked decline in surgical intervention with advancing age, from 46% in patients <65 years to only 6% in those ≥80 years. Among patients aged ≥75 years, surgical management remained associated with favorable long-term survival outcomes (HR 0.36; 95% CI 0.24–0.54; *p* < 0.001) [12].

### 2.2. Chronic Kidney Disease (CKD) and ESRD Requiring Renal Replacement Therapy (RRT)

ESRD requiring RRT has long been identified as a risk factor for the development of IE. The incidence ranges from 1.7 to 2 cases per 1000 patients and may be up to 70 times greater than the reported incidence in patients without CKD [18,19,20]. In an international prospective cohort study encompassing *7715* IE episodes, 8.3% of patients were on hemodialysis (HD) [21], a slightly higher rate than the previously reported prevalence of 2–6% [18,19,20,22,23,24]. Accordingly, HD represents one of the strongest healthcare-associated risk factors for IE [21].

Predisposing factors for the occurrence of IE following RRT include the type of vascular access used for dialysis [18,20,21], the CKD-associated immune dysfunction [25], and coexisting conditions [20,21,26,27], such as diabetes mellitus, chronic cardiovascular diseases and protein malnutrition. Both the modality of RRT and the type of hemodialysis access have been shown to influence the incidence of bacteremia and, consequently, the risk of IE [20,21]. Data from the Danish National Patient Registry demonstrated a higher incidence of IE among patients receiving HD through central venous catheters (CVCs), with rates up to 3.5-fold greater than those observed among patients using arteriovenous (AV) fistulas [20]. Similar findings have been reported in other studies, showing that temporary vascular access and indwelling HD catheters increase the risk of life-threatening bacteremia and, consequently, IE [28,29,30,31], compared with AV grafts and native AV fistulas [32,33].

Recent registry-based data have provided further insight into the clinical profile of patients with ESRD who develop IE. A nationwide Spanish epidemiological study showed that, compared with non-ESRD patients, those with ESRD were younger (*p* < 0.001) yet had a substantially greater comorbidity burden (CCI > 2: 76.4% vs. 32.2%; *p* < 0.001). ESRD patients also exhibited fewer PVE but a higher prevalence of CIED-IE, together with a distinct microbiological profile characterized by more frequent staphylococcal infections, including both *S. aureus* and CoNS, whereas viridans group streptococci predominated among non-ESRD patients. Within the ESRD population, dialysis-dependent patients were younger and more comorbid, with a higher prevalence of CIED-IE. Moreover, *S. aureus* predominated in dialysis patients, whereas enterococci were more frequently identified in non-dialysis patients [26]. Consistent results have been observed in Danish nationwide registry analyses by Stahl and colleagues, comparing patients with IE, stratified by hemodialysis status [27].

The prognosis of IE in patients with ESRD remains poor. Approximately one-third of patients die during hospitalization [18,21,26,27], and about half survive in the short-term [34]. Despite conflicting evidence regarding in-hospital mortality, longer-term outcomes appear consistently worse in patients receiving hemodialysis, with higher mortality observed at both 1 and 5 years [27,35].

### 2.3. Underlying Medical Conditions

An immunocompromised state resulting from conditions such as HD, malignancy, autoimmune disorders, other chronic diseases, or the use of immunosuppressive agents (e.g., immunotherapy and immunomodulatory treatments) is a well-known risk factor for IE, even in the absence of valvular abnormalities [36]. However, it remains to be elucidated whether immunosuppression independently contributes to the overall risk of IE or constitutes one element in the multi-step process required for the development of IE in patients with other predisposing factors, including frequent healthcare exposure and presence of indwelling catheters.

#### 2.3.1. Solid Organ Transplantation

Τhe epidemiology and clinical impact of IE among SOT recipients remain poorly characterized, with available evidence largely limited to small case series and single-center retrospective studies. As expected, the prevalence of IE in solid organ transplant (SOT) recipients is higher than in the general population, and nosocomial or healthcare-acquired cases are most implicated [37,38]. Based on a retrospective analysis of 2016–2019 United States National Inpatient Sample database, the prevalence of SOT was 0.6% among index hospitalization for IE, of which the most prevalent SOT was kidney transplant (0.4%), followed by heart or lung (0.1%), liver (0.1%), pancreas (0.04%), and intestine transplant (0.01%) [39]. Remarkably, kidney transplant recipients account for approximately 70% of all SOT patients who develop IE [39,40]. This likely reflects the increased prevalence of surviving kidney transplant recipients as compared to recipients of other solid organs [41], the higher risk of infection rising from retained permanent HD access (e.g., AV grafts) [42], and the valvular calcification frequently observed in population with ESRD [43].

According to the most recent studies, SOT-IE is associated with an in-hospital mortality rate as high as 30.6%. Still, no significant differences in mortality have been recorded in patients with and without SOT [38,39,40]. This may be explained by the fact that: (i) there was a relatively lower number of SOT recipients (<2%) included in the studies and/or (ii) this frail and high-risk patient group is routinely followed by a dedicated SOT-team, thereby facilitating earlier, prompt diagnosis, particularly in cases of nosocomial- or healthcare-associated IE.

#### 2.3.2. Cancer

IE is increasingly recognized as a significant complication in the oncological population. Reported prevalence rates vary considerably across cohorts, ranging from 5.6% to 18% [44,45], and patients with intestinal cancers and lymphoma are predominantly affected [45,46].

Historically, cancer-related IE has been primarily attributed to the immunocompromised milieu inherent to malignancy, tumor-related hypercoagulability, and the presence of indwelling devices such as CVCs or pacemaker (PM) leads [45,47]. However, most recent evidence suggests that comorbidities (e.g., obesity), recent surgical interventions, and invasive procedures may exert a more pronounced causal influence than the underlying malignancy itself [48], underscoring the dynamic interplay between host vulnerability, healthcare exposure, and iatrogenic risk factors.

Lastly, cancer-related IE has been linked with higher short- and long-term mortality compared to non-cancer patients [44,45,46], with in-hospital and one-year mortality reaching approximately 35% and 48%, respectively. Furthermore, IE often necessitates postponement or discontinuation of cancer therapy, thereby compromising overall oncologic outcomes.

#### 2.3.3. Autoimmune Disorders

Contemporary evidence continues to support a higher susceptibility to IE among patients with autoimmune diseases, including inflammatory bowel disease, systemic lupus erythematosus (SLE), and anti-phospholipid syndrome (APS) [49,50]. While the underlying mechanisms have not been elucidated, these patients represent a high-risk population for opportunistic and healthcare-associated infections owing to receipt of prolonged use of corticosteroids or immunomodulatory therapies, repeated exposure to healthcare settings, and the need for invasive medical procedures that predispose them to transient bacteremia. For instance, in a Taiwan-based nationwide cohort of more than 12,000 patients with SLE, the incidence of IE was nearly 10-fold higher than in matched controls (*p* < 0.001), with pre-existing heart disease, CKD, recent dental procedures, and intravenous corticosteroid therapy identified as independent risk factors [51]. Similarly, in a large cohort of nearly 300,000 patients, APS was independently associated with more than a 2-fold increase in the odds of IE, as well as a markedly higher risk of MRSA and methicillin-susceptible *S. aureus* (MSSA) sepsis, underscoring a broader infection susceptibility likely related to endothelial dysfunction and immune dysregulation inherent to APS [52].

### 2.4. IDU

People who inject drugs (PWID) are thought to have an up to 100-fold increased risk of IE relative to the general population, and several mechanisms are implicated in the pathogenesis of IDU-IE, including endothelial damage caused by the injection of particulate matter, use of contaminated equipment and non-sterile injection techniques, and drug-associated vasospasm leading to intimal damage and thrombus formation [53].

The incidence of IE closely parallels epidemiological trends in IDU [54,55,56,57], with notable geographic variations. Analysis of US population-based data over a 5-year period demonstrated that IDU-IE accounted for 22.2% of IE cases, with its incidence nearly doubling from 15% in 2010 to 29% in 2015 [53]. In contrast, data from the EURO-ENDO registry reported a history of IDU in only 6.9% of patients with IE, highlighting substantial regional differences [9].

The observed differences may, in part, reflect the opioid epidemic in the US, given the strong association between opioid use and IDU-IE [58,59]. The sustained rise in IDU-IE has also contributed to a marked increase in IE-related hospitalizations. Between 2000 and 2013, hospitalizations attributable to IDU-IE increased by 238% [56], a trend further confirmed in more recent analyses reporting up to a 12-fold increase between 2007 and 2017 [60].

The demographic and clinical profile of patients with IDU-IE is well-characterized and differs from that of patients with IE due to other causes. Patients with IDU-IE are consistently typically younger and have fewer comorbidities than those with non-IDU-IE [59,60,61]. However, a history of prior IE, as well as comorbid HIV infection and chronic liver disease, is frequently observed [59,61,62]. Although IDU-IE predominantly affects the native tricuspid valve [62], recent data from Pericàs and colleagues found that left-sided involvement may also be substantial, with approximately half of IDU-IE cases involving the left-sided valves and 34.5% presenting exclusively as left-sided IE [61].

Younger age and a lower burden of comorbidities do not translate into favorable long-term outcomes in PWID with IDU-IE [62,63]. The unfavorable outcomes are largely driven by continued drug use, a predilection for recurrent endocarditis, and the development of new sites of infection [64]. A recent large cohort study comparing IDU-IE with non-IDU-IE showed poorer outcomes and recurrent IE when patients with IDU-IE were treated with medical therapy alone, and a steady increase in the need for surgical management in this patient population. Furthermore, lower short-term mortality (6.8% vs. 9.6%, *p* < 0.001) was observed in IDU-IE patients compared with non-IDU-IE patients [53]. Lastly, in an observational cohort study of PWID hospitalized with either IE or other infections, long-term survival was significantly lower among those with IE. Half of the deaths in this group were attributed to either a relapse or recurrent episodes of endocarditis [65], thus suggesting that adverse outcomes may be linked to ongoing infection [66].

## 3. Healthcare-Associated Risk Factors

The increasing use of prosthetic cardiac material has substantially altered the epidemiology of IE. Whereas early studies reported PVE in approximately 5% of all IE cases, nowadays it accounts for up to 20–30% of cases [9,67]. Despite advances in diagnosis and management, PVE is accompanied by substantial morbidity and mortality, with in-hospital mortality rates of up to 27% and 1-year mortality approaching 37% [67]. Beyond conventional PVE, advances in cardiovascular medicine have introduced newer device-associated forms of IE, including TAVR-, TPVR-, and CIED-associated infections, each characterized by distinct epidemiological, microbiological, diagnostic, and clinical features.

### 3.1. TAVR

Reported incidence rates of TAVR-IE vary across studies, ranging from 0.3 to 2 cases per 100 person years (py) [68]. A large U.S. nationwide retrospective analysis of administrative data reported an annual incidence of 0.87% [69], whereas a multicenter observational study found comparable incidence rates between earlier (pre-2014) and more contemporary (post-2014) procedural eras (5.45 vs. 6.52 per 1000 person-years; *p* = 0.12) [70]. Similar estimates have been described in other large cohorts, including an incidence of 1.1% per person-year in an international study of 20,006 patients [71] and 1.89% in a nationwide French population of 107,786 patients with aortic stenosis [72]. Overall, this variability likely reflects differences in patient populations, procedural eras, and case definitions, including the inclusion of both possible and definite TAVR-IE in some analyses.

The temporal distribution of TAVR-IE remains incompletely defined, with studies reporting heterogeneous patterns over time. Nevertheless, most cases appear to occur within the first year following valve implantation, with incidence rates generally declining thereafter [69,73,74]. Data from the SwissTAVI Registry showed the highest incidence during the peri-procedural phase (2.59 per 100 py), with progressively lower rates during later follow-up (delayed-early: 0.71 per 100 py; later: 0.4 per 100 py) [73]. Similar observations were reported in U.S. and Swedish nationwide cohorts [69,74], whereas smaller studies involving lower-risk populations suggested a more delayed occurrence of TAVR-IE, with incidence rates increasing beyond the early post-procedural period (≤30 days: 0%; 31–365 days: 1.5%; >1 year: 2.8%) [75]. Interestingly, recent cohorts appear to demonstrate lower rates of early post-procedural IE, possibly reflecting advances in procedural techniques and patient selection [70]. Available comparative studies evaluating TAVR and SAVR have reported largely comparable PVE rates, albeit with some variability. A meta-analysis of 10 randomized clinical trials (RCTs), including over 9200 patients, showed no difference in the incidence of IE between TAVR and SAVR at 30-day and 1-year follow-up [76], consistent with several observational studies [72,77,78,79]. However, longer-term analyses have yielded conflicting results, with some studies reporting a higher cumulative incidence following SAVR [80,81], whereas a Swiss nationwide cohort demonstrated an increased risk after TAVR compared with bioSAVR (HR 1.56; 95% CI 1.12–2.18) [82].

The diagnosis of TAVR-IE remains difficult, and identifying patients at higher risk is of utmost importance. Several risk factors for TAVR-IE have been identified, including male sex; cardiovascular or other chronic diseases (e.g., CKD, diabetes mellitus, and cancer); paravalvular leakage; new pacemaker implantation; orotracheal intubation; lack of pre-dilatation; and an elevated EuroSCORE II [69,70,83]. Although IE is most common in older patients, younger age was an independent risk factor for TAVR-IE [79], reflecting the severe comorbidity burden that may have led to younger patients being selected for TAVR.

TAVR-IE has distinct microbiological characteristics that should be recognized. Notably, up to half of cases are healthcare-associated, thereby increasing the likelihood of antimicrobial resistance [68,71]. In contrast to NVE, PVE, and SAVR-IE, where *S. aureus* and other staphylococci predominate, TAVR-IE is most commonly caused by enterococci, followed by *S. aureus* and CoNS. Enterococci account for more than one-quarter of TAVR-IE cases, potentially related to the frequent use of the transfemoral approach and increased exposure to groin flora [84].

Common sources of bacteremia include intravascular and soft tissue infections, as well as gastrointestinal and urological foci; however, in nearly 70% of cases, no source is identified despite thorough investigation [68]. Furthermore, TAVR-IE engenders unfavorable outcomes, with up to two-thirds of patients developing at least one complication. In-hospital mortality for patients with TAVR-IE ranges from 16% to 36% and may reach up to 59% at 1-year follow-up [85].

Even though surgery remains a cornerstone of IE management, its role in TAVR-IE is only partially addressed in current guidelines, with decisions often individualized based on local expertise, surgical risk, and expected outcomes with medical therapy alone [85]. A recent meta-analysis including 1557 patients found no significant difference in short-term mortality between surgical and medical management (9.7% vs. 8.4%), although the results were limited by substantial heterogeneity and wide confidence intervals [86]. In contrast, Fukuhara and colleagues reported markedly different outcomes among 67 patients with TAVR-IE. Notably, 44.2% of medically managed patients had a guideline-based indication for surgery but did not undergo intervention. Mortality was highest among these patients, reaching 31.6% at 30 days and 73.7% at 1 year [87]. However, larger observational cohorts, including the Infective Endocarditis after TAVI International Registry and the U.S. Nationwide Readmission Database, failed to demonstrate a significant mortality benefit associated with surgical intervention [88,89]. These discepancies may be attributed, at least in part, to earlier surgical intervention and greater institutional experience with post-TAVR reoperations in the study by Fukuhara and colleagues [87].

### 3.2. Transcatheter Pulmonary Valve Replacement (TPVR)

The risk of IE following TVPR is not negligible, with an estimated incidence of 16–27 per 1000 py [90], potentially threatening long-term valve function. Multiple factors contribute to the risk of TPVR-associated IE, including host-related characteristics (e.g., age, sex, genetic syndromes, immunosuppression, and prior IE), device- and procedure-related characteristics (e.g., valve type, elevated residual transpulmonary gradients), and microbial factors related to the portal of pathogen entry and specific pathogen characteristics [90].

One of the most extensively studied risk factors is the relationship between valve type and the occurrence of IE. The two valves approved for TVPR are the Melody Medtronic (Medtronic Inc., Minneapolis, MN, USA) [91] and the Edwards Sapien (Edwards Lifesciences, Irvine, CA, USA) [92]. So far, the Melody valve has been associated with a higher pooled incidence of IE, ranging from 4.9% to 8.5%, compared with the Sapien valve, which has a pooled incidence of 1.3% to 3.1% [93,94], largely attributable to specific manufacturing features. Additionally, the use of bovine jugular grafts has been identified as an independent risk factor for IE, conferring a markedly higher risk compared with homografts (HR 9.05; 95% CI 2.6–31.8) [95]. IE after implantation of the Melody valve is not limited to the early post-procedural period, but has a median time to diagnosis of 18 months [96], suggesting that risk persists over time rather than being driven solely by procedural factors. This most likely reflects the prosthetic material as a risk factor [97]. In fact, more than half of affected patients require reintervention, while mortality has been reported at approximately 8.7% [96].

### 3.3. CIED

CIEDIs present with different clinical scenarios, from pocket infection (PI) to IE, and they are burdened by high morbidity, mortality, and costs [98]. It is noteworthy that not all patients with a CIED and positive blood cultures have an underlying CIED lead infection, and the risk is determined by various factors, including the duration and source of bacteremia, device type, number of device-related procedures, and the type of microorganism isolated [99].

CIEDIs have also shown an upsurge over the years, and their incidence ranges from 0.5% to 2.2% across different populations, device types, and time from implant [100]. Notably, CIEDI’s rate increase has surprisingly exceeded the rise in device implantations [99]. The escalation is probably attributed to the frailty of CIED recipients in terms of age and debilitating comorbidities [98,99,100,101], as well as to procedure-related factors (e.g., CIED type and complexity, techniques, prolonged procedural time, early re-intervention) [98,102,103,104]. Özcan and colleagues analyzed all de novo permanent pacemakers (PMs) and implantable cardioverter-defibrillators (ICDs) together with the occurrence of post-implantation IE-events in the period from 2000 to 2012 based on Danish nationwide administrative registers. They concluded that IE incidence increased with device complexity, as a dose-response relationship between the number of leads and IE incidence was observed, and that it was particularly high during the first year. Specifically, the incidence (per py) in PM was 2.1% for single-chamber devices and 6.2% for CRT, and PM complexity was an independent risk factor for IE. Similarly, the IE rate in ICDs was 3.7% in single-chamber devices and 6.3% in CRT [103]. In tandem, early-onset IE following CIED implantation has been described in several studies [105,106,107,108], and is attributed, to some extent, to contamination at the time of implantation.

The microbiology of CIEDIs is dominated by Gram-positive pathogens, particularly *S. aureus* and CoNS, reflecting the importance of skin colonization, procedural contamination, and biofilm formation on device surfaces [109,110]. Contemporary cohorts have documented a substantial increase in the burden of CIED-IE over recent decades, accompanied by a shift toward older patients with a greater comorbidity burden and increasingly complex cardiac devices [111]. The presence of infected hardware has important therapeutic implications, and current consensus documents recommend complete device extraction whenever feasible, as retention of infected material has been associated with persistent infection, recurrent bacteremia, relapse, and worse clinical outcomes [109,110,111].

Mortality after CIED infection is high and appears to be greater in the case of IE relative to PI [104]. The overall mortality ranges from 4 to 36%, the in-hospital mortality from 9.8 to 33.3%, and the 10-year mortality from 53.3 to 80% [112]. At the same time, left-sided endocarditis and CDRIE removal/reimplantation are coupled with a worse prognosis within the first year [113].

## 4. Microbiological and Resistance Trends

The microbiological profile of IE closely reflects the broader epidemiological transition of the disease. The growing predominance of healthcare-associated IE, largely caused by staphylococci and enterococci with the former pathogen accounting for almost one third of contemporary cases, has heightened concerns regarding antimicrobial resistance, particularly given the increasing prevalence of methicillin- and vancomycin-resistant strains [6,114,115,116,117]. The increasing importance of enterococci represents another hallmark of the contemporary microbiology of IE. *E. faecalis* has emerged as a major pathogen in healthcare-associated IE and PVE, particularly among older patients with multiple comorbidities and frequent healthcare exposure. This shift is especially evident in TAVR-IE, where enterococci have emerged as the predominant causative pathogens in contemporary cohorts [84]. Reflecting its growing epidemiological and clinical significance, *E. faecalis* is recognized as a typical microorganism causing infective endocarditis in the 2023 Duke-ISCVID diagnostic criteria [118]. On the other hand, streptococci now represent less than 20% of the total burden of the disease [115]. Despite emerging resistance among Gram-positive pathogens involved in IE, the therapeutic component seems to be enriched by the addition of long-acting antibiotics and the shortening of the previously mandatory daily iv antibiotic administration [85]. Nevertheless, current epidemiological data show that antimicrobial resistance has important prognostic implications. A recent nationwide population-based study from Spain showed that antimicrobial-resistant IE accounted for 7.64% of bacterial IE hospitalizations and was associated with significantly higher in-hospital mortality compared with non-resistant infections (29.36% vs. 17.68%), corresponding to a 39% increase in mortality risk [119]. Furthermore, Gram-negative IE although encountered less frequently than Gram-positive disease, is associated with unfavorable clinical outcomes, especially in cases involving carbapenem-resistant pathogens [114,119,120,121]. For instance, in one study, more than one-third of Gram-negative isolates exhibited resistance to at least three antibiotics, while patients with Gram-negative IE had a more than fourfold higher risk of mortality [122]. At the same time, fungal endocarditis, predominantly caused by *Candida* and *Aspergillus* species, remains an uncommon but highly lethal entity, accounting for approximately 2% of IE cases and occurring mainly in immunocompromised patients and recipients of intravascular or intracardiac devices [123]. Taken together, these observations highlight the growing complexity of contemporary IE, where resistant and opportunistic pathogens increasingly compromise empiric treatment strategies and contribute to adverse clinical outcomes.

## 5. Conclusions

The changing epidemiology of IE mirrors broader shifts in modern medicine and population health. Increased life expectancy, the expanding use of invasive cardiac procedures and devices, and the growing burden of chronic diseases have altered not only the populations affected by IE, but also the way the disease presents in clinical practice. In the modern world, IE may be viewed as a consequence of advances in medical care, with a growing proportion of cases occurring in patients following medical interventions rather than in those with underlying cardiac conditions [124].

These developments highlight the increasingly complex nature of IE and the need for approaches that extend beyond antimicrobial treatment alone. Earlier recognition, coordinated multidisciplinary care, and preventive strategies tailored to vulnerable populations are likely to become increasingly important in the years ahead. Equally important is the need to improve access to specialized diagnostic and surgical care, particularly in resource-limited settings. The need of surgical intervention is often hampered by the underlined comorbidities of the host, making adequate surgical treatment an essential challenge in the contemporary management of IE [125,126]. Nevertheless the rapid expansion of percutaneous techniques may provide bridging or even definitive solutions in high surgical risk patients [127]. Continued epidemiological surveillance and a better understanding of emerging risk profiles will remain essential for improving outcomes and shaping future clinical practice.

## Data Availability

No new data were created or analyzed in this study.
